# Widely targeted metabolomic profiling combined with transcriptome analysis sheds light on flavonoid biosynthesis in sweet orange 'Newhall' *(C. sinensis)* under magnesium stress

**DOI:** 10.3389/fpls.2023.1182284

**Published:** 2023-05-12

**Authors:** Bo Xiong, Qin Li, Junfei Yao, Zhuyuan Liu, Xinxia Yang, Xiaoyong Yu, Yuan Li, Ling Liao, Xun Wang, Honghong Deng, Mingfei Zhang, Guochao Sun, Zhihui Wang

**Affiliations:** College of Horticulture, Sichuan Agricultural University, Chengdu, China

**Keywords:** *Citrus sinensis*, flavonoid biosynthesis, magnesium stress, flavones, CHS, regulatory mechanism

## Abstract

Sweet orange ‘Newhall’ peels (SOPs) are abundant in flavonoids, making them increasingly popular in the realms of nutrition, food, and medicine. However, there is still much unknown about flavonoid components in SOPs and the molecular mechanism of flavonoid biosynthesis when subjected to magnesium stress. The previous experiment conducted by the research group found that the total flavonoid content of Magnesium deficiency (MD) was higher than Magnesium sufficiency (MS) in SOPs. In order to study the metabolic pathway of flavonoids under magnesium stress, an integrative analysis of the metabolome and transcriptome was performed in SOPs at different developmental stages, comparing MS and MD. A comprehensive analysis revealed the identification of 1,533 secondary metabolites in SOPs. Among them, 740 flavonoids were classified into eight categories, with flavones identified as the dominant flavonoid component. The influence of magnesium stress on flavonoid composition was evaluated using a combination of heat map and volcanic map, which indicated significant variations between MS and MD varieties at different growth stages. The transcriptome detected 17,897 differential genes that were significantly enriched in flavonoid pathways. Further analysis was performed using Weighted gene correlation network analysis (WGCNA) in conjunction with flavonoid metabolism profiling and transcriptome analysis to identify six hub structural genes and ten hub transcription factor genes that play a crucial role in regulating flavonoid biosynthesis from yellow and blue modules. The correlation heatmap and Canonical Correspondence Analysis (CCA) results showed that *CitCHS* had a significant impact on the synthesis of flavones and other flavonoids in SOPs, as it was the backbone gene in the flavonoid biosynthesis pathway. The qPCR results further validated the accuracy of transcriptome data and the reliability of candidate genes. Overall, these results shed light on the composition of flavonoid compounds in SOPs and highlight the changes in flavonoid metabolism that occur under magnesium stress. This research provides valuable insights for improving the cultivation of high-flavonoid plants and enhancing our understanding of the molecular mechanisms underlying flavonoid biosynthesis.

## Introduction

Citrus fruits, members of the Rutaceae family, are widely consumed throughout the world. A prime example is the sweet orange ‘Newhall’ (*C. sinensis*), which stands out for its exceptional quality and has its origins in America. Citrus fruits are a rich source of bioactive flavonoids, with the peels often containing a higher concentration of these compounds than pulp and seeds (Li et al., 2022a; Yu et al., 2022). Previous studies have shown that flavonoid compounds in Citrus peel play a significant role in anti-inflammation, anti-oxidation, immune regulation, and prevention and treatment of multiple respiratory diseases (Peng et al., 2019; Singh et al., 2020). Unlike most other fruits, Citrus species mainly accumulate flavonone glycosides and polymethoxylated flavones (PMFs) as their main flavonoids (Zhao et al., 2021). These compounds are highly valued as a source of common Chinese medicines, food, and nutritional supplements due to their abundance of bioactive components.

Flavonoids are a significant group of secondary polyphenolic metabolites with a chemical structure of 3-C (C6-C3-C6) (Li et al., 2020b; Nabavi et al., 2020). These compounds are widely distributed throughout the plant kingdom, and more than 6,000 distinct flavonoids have been identified to date (Lepiniec et al., 2006; Ferrer et al., 2008). The pathways involved in flavonoid metabolism have been extensively studied in model plants (Deng and Lu, 2017; Tohge et al., 2017). The flavonoid biosynthesis process begins with the primary glucose produced through photosynthesis, which is then converted into phenylalanine through glycolysis, pentose phosphate, and shikimic acid pathways. Phenylalanine enters the phenylpropane metabolic pathway through the action of phenylalanine ammonylase (PAL). In this pathway, phenylalanine is converted into p-coumaryl CoA through a series of reactions catalyzed by PAL, cinnamate 4-hydroxylase (C4H), and 4-coumaroyl CoA ligase (4CL) (Forkmann and Martens, 2001; Du et al., 2010; Saito et al., 2013). The flavonoid biosynthesis pathway is initiated by condensation of one molecule of 4-coumaroyl-CoA with three molecules of malonyl CoA by chalcone synthase (CHS) enzyme. Subsequently, chalcone isomerase (CHI), flavanone 3-hydroxylase (F3H), dihydroflavonol 4-reductase (DFR), and anthocyanidin synthase (ANS) lead to the synthesis of anthocyanidin pigments. Flavone synthase (FNS) and isoflavone synthase (IFS) produce flavones and isoflavones, respectively. Flavonol synthase (FLS) catalyzes dihydroflavonols to flavonols, while leucoanthocyanidin reductase (LAR) and anthocyanidin reductase (ANR) synthesize cis- or trans-flavan-3-ols, which are precursors of proanthocyanidin (PA) polymers (Owens et al., 2008; Li et al., 2020a). Regulation of genes involved in flavonoid biosynthesis is controlled through specific mechanisms that vary depending on the species or tissue involved (Zhang et al., 2022). The accumulation of flavonoids is mainly controlled by structural biosynthetic genes, regulatory MYB transcription factors (TFs) and the MYB-bHLH-WD40 (MBW) complex (Appelhagen et al., 2011; Hichri et al., 2011; Song et al., 2019; Yang et al., 2023). These factors are responsible for regulating the expression of genes involved in flavonoid biosynthesis, leading to their accumulation.

To date, efforts to identify the chemical compositions of citrus, especially flavonoid compounds, have increased significantly (Barreca et al., 2017; Mahmoud et al., 2019; Yu et al., 2022). However, only a handful of flavonoid components present in citrus, specifically in SOPs, have been successfully identified. This limitation may hinder the growth of SOPs utilization in the food industry. Furthermore, flavonoids are a diverse group of plant metabolites that have diverse structures, wide distribution, and critical roles in plant growth, adaptation, signaling, and response to biotic and abiotic stresses (Winkel-Shirley, 2001; Treutter, 2005; Rowan et al., 2009; Misra et al., 2010; Zhang et al., 2019). When the environment changes, flavonoid levels may also change. For example, Zanthoxylum bungeanum cv. “Fengjiao” exhibited an increase in total flavonoid content under drought stress (Hu et al., 2021), while high solar radiation led to an increase in flavonol content in Ginkgo biloba (Guo et al., 2020). In China, where citrus is mainly grown in subtropical and tropical regions, soil acidification and consequent magnesium (Mg) leaching are major problems (Long et al., 2017). In addition, improper use of chemicals and inadequate use of organic fertilizers and medium and trace element fertilizers can lead to Mg deficiency in citrus, resulting in a decline in fruit quality and yield. Interestingly, Mg deficiency was found to increase total flavonoid content (Ramakrishna and Ravishankar, 2011; Bartwal et al., 2013; Li et al., 2017; Khare et al., 2020). However, the underlying mechanism remains unexplored in SOPs.

In recent years, analytical methods such as multi-omics have been widely used in the study of food components and functions. To investigate flavonoid compositions in SOPs and elucidate the regulatory mechanism of flavonoid biosynthesis under magnesium stress, transcriptomic and metabolomic analyses were performed. Through these analyses, six hub candidate structural genes and ten hub TF genes involved in flavonoid biosynthesis regulation were identified using WGCNA and CCA. This valuable information enhances our understanding of the nutritional value of SOPs and provides insights into their potential use in food.

## Materials and methods

### Plants and sample preparation

**Figure 1:**
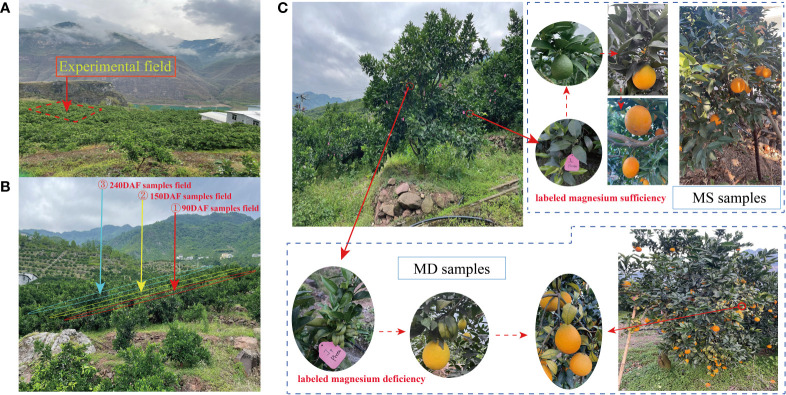
Experimental environment and design. **(A)** Experimental field. **(B)** Sample collection field at 90DAF, 150DAF and 240 DAF groups. **(C)** Methods for collecting samples of MS and MD fruits.

Sweet orange ‘Newhall’ cv. C. retuculata Blanco plants were cultivated in Leibo County, Liangshan Prefecture, Sichuan Province, China (30.27°N, 120.20°E). For the experiment, 13-year-old trees were selected that exhibited uniform growth and were divided into three groups based on their stage of development: the young fruit stage in June (90 days after flowering, 90DAF), the rapid expansion stage in August (150 days after flowering, 150DAF), and the fruit maturation stage in November (240 days after flowering, 240DAF) sample fields (**Figures 1A, B**). Since MS and MD samples were collected from different branches of the same tree, all samples were labeled in March 2022 (**Figure 1C**). At 90DAF, MS samples were collected from branches without symptoms of magnesium deficiency, while MD samples were collected from branches with leaf yellowing due to magnesium deficiency. Samples were collected at 150DAF and 240DAF consistent with 90DAF. Citrus fruits were collected without any damage, pests, or diseases. MS fruits were collected during three phenological periods, namely, MS1, MS2, and MS3, representing 90DAF, 150DAF, and 240DAF, respectively. Similarly, MD fruits were collected at the same three stages, namely, MD1, MD2, and MD3, representing 90DAF, 150DAF, and 240DAF. Eighteen trees were selected for each period. The pulp and peel of fruits were separated, cut, frozen and stored in a -80°C refrigerator for flavonoid metabolite determination and transcriptome sequencing.

### Measurement of total flavonoids, MDA content and SOD activity

An improved protocol was used to determine the total flavonoid content of citrus peels (Wang et al., 2007; Yu et al., 2022). Initially, 0.5 g citrus peel powder was weighed and dissolved in 10 mL 70% absolute ethanol at a ratio of 1:20 (w/v). The mixture was then subjected to ultrasonic treatment at 55°C for 40 min, followed by filtration. To 1 mL of the extraction solution, 0.5 mL of 5% NaNO_2_ solution was added sequentially and well shaken. The mixture was then left for 5 min. Next, 0.5 mL 10% Al(NO_3_)_3_ was added to the solution, mixed thoroughly, and left for 6 min. Finally, 5 mL of 1mol/L NaOH was added, and distilled water was added up to 10 mL. The mixture was shaken and left for 10 min to complete the reaction. The absorbance value of solution was measured at 510 nm, with rutin (purity≥98%, sourced from Leaf Shanghai Biological Technology Co., Ltd.) used as the standard product. The flavonoid content (U mol/g) was calculated using the formula (C*V)/W, where C represents the concentration, V represents the volume, and W represents the weight of the sample. To determine the MDA content and SOD activity, Li’s method was followed (Li et al., 2022b).

### Metabolomic profile detection and analysis

The samples underwent a series of preparation steps, including immersion in liquid nitrogen, grinding to a fine powder, suspension in 70% methanol and centrifugation at 20,000 rpm for 20 minutes at 4°C. The supernatant was then filtered through a 0.22-μm nylon syringe filter before being subjected to UPLC-MS analysis. This involved the use of an Agilent SB-C18 column (2.1 × 100 mm, 1.8 μm), with mobile phase A consisting of water with 0.1% formic acid, while mobile phase B was a solution of 0.1% formic acid in acetonitrile. The elution procedure utilized a gradient of 0-95% B from 0-9 min, followed by a single minute at 95% B and further gradient steps of 95-5% B for 1 minute and 5% B from 11-14 minutes. The flow rate of the system was set at 0.35 mL/min, and the column temperature was held constant at 40°C, with an injection volume of 4 μL. Multiple reaction monitoring (MRM) mode was used to acquire data from the production scan, which was then analyzed with the Metabolites Database (METWARE database) for metabolite identification. Quantitative analysis of metabolites was then performed using Analyst 1.6.3 software, with further examination of the metabolic pathways of these compounds using the KEGG database (http://www.kegg.jp).

### Transcriptome analysis

RNA isolation and sequencing were performed by Metware Biotechnology Co., Ltd. (Wuhan, China). The quality of the cDNA libraries was examined, and PCR amplification and sequencing were performed using an Illumina HiSeq™ 2500 platform. The obtained reads were processed to remove contaminants and mapped to the reference genome sequence of C. sinensis v3.0 (http://www.hzau.edu.cn) using HISAT 2.2.4. StringTie was used for transcript assembly. Fragments per kilobase per million (FPKM) values were calculated to determine gene abundance and normalize data. Differential expression analysis of mRNAs was performed using DESeq2 software. DEGs were identified based on fold change > 2 and false discovery rate (FDR) < 0.05 as cutoff values (P < 0.05).

### Weighted gene co-expression network analysis

In order to construct the gene coexpression network, the WGCNA package was utilized with a soft-thresholding power β of 20 (Langfelder and Horvath, 2008), and the resulting modules in different colors were obtained through the DynamicTreeCut algorithm. The coexpression networks were visualized using Cytoscape software version 3.9.1, with the selection of specific genes. The KEGG database was further applied to enrich the metabolic pathways of the chosen modules.

### Principal component analysis and canonical correlation analysis

To analyze the relative levels of 740 flavonoids and the expression levels of differentially expressed genes in the transcriptome, principal component analysis (PCA) was performed using the default parameters of the Metware data processing platform (https://www.metware.cn/). Canonical correlation analysis (CCA) was conducted to explore the potential correlation between flavonoid metabolite levels and synthetic genes through metabolomic and transcriptomic data. Specifically, 21 flavonoid content levels and the expression levels of 41 hub genes within the flavonoid synthesis pathway of MS and MD peels at various stages were imported into Canoco 5.0 for CCA analysis, utilizing the default parameters.

### Quantitative real-time PCR analysis

First-strand cDNA was synthesized from each RNA sample (0.2 μg). Specific primers (**Supplementary Table S1**), designed by NCBI Primer-BLAST using genome sequences, were used for quantitative real-time PCR (qPCR) cycling on a CFX96 Real-Time PCR Detection System. Real-Time PCR System (Hercules, CA, USA). The qPCR cycling conditions were 95°C for 2 minutes followed by 39 cycles of 95°C for 5 seconds and 57°C for 40 seconds. Actin was utilized as an internal control (**Supplementary Table S1**), and biological replicates were triplicated.

### Statistical analysis

All tests were performed three times, and the results were presented as the mean value ± standard deviation. Significant differences were analyzed using SPSS Statistics 24 software (IBM Inc., Chicago, IL, USA) with Duncan’s test. The heatmap was created using the heatmap2 feature of the R package.

## Results

### Total flavonoid and MDA content, SOD activity in SOPs

**Figure 2:**
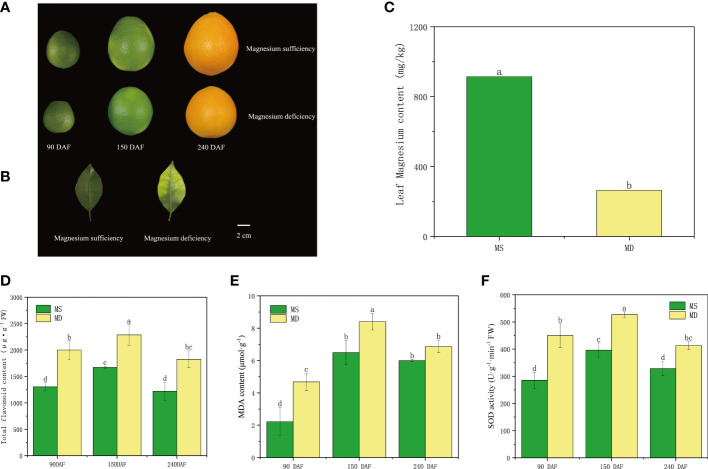
Changes in flavonoid compositions of SOPs under magnesium stress. **(A, B)** The phenotype of Magnesium sufficiency and Magnesium deficiency in SOPs and leaves. **(C)** The Magnesium content of leaves between Magnesium sufficiency and Magnesium deficiency. **(D)** The relative content of total flavonoid of SOPs from MS and MD. **(E, F)** The relative content of MDA and SOD activity of SOPs from MS and MD under magnesium stress. Values in each column with different letters are significantly different at *P*<0.05.

Fruits and leaves of MS and MD were collected at the three stages for analysis (**Figures 2A, B**). MD leaves showed yellowing in the mesophyll, with veins remaining green in an inverted V shape. In contrast, MS leaves retained their green color throughout the sampling period. MD leaves showed magnesium deficiency, and analysis indicated that the magnesium content in MD leaves was 264.16 mg/kg, which was lower than the optimal magnesium content. In contrast, the magnesium content in MS leaves was 914.28 mg/kg, which was higher than the appropriate magnesium content range (**Figure 2C**). To gain a better understanding of the impact of magnesium deficiency on SOPs, total flavonoid content, MDA content, and SOD activity were analyzed (**Figures 2D–F**). The results showed that the total flavonoid content in MS increased from 1303.16 μg/g to 1670.58 μg/g during the young fruit stages, and then decreased to 1220.46 μg/g during fruit maturation stage. In MD, flavonoid content increased from 2000.38 μg/g to 2285.73 μg/g, before decreasing to 1826.01 μg/g. Total flavonoid content in MD was consistently higher than that of MS at all three stages. Flavonoid compounds act as antioxidants and play a vital role in maintaining cellular function under magnesium deficiency stress. The MDA content and SOD activity of both MS and MD mirrored the trend for total flavonoid content at different developmental stages (**Figures 2E, F**).

### Metabolome profiling in SOPs at different development stages

**Figure 3:**
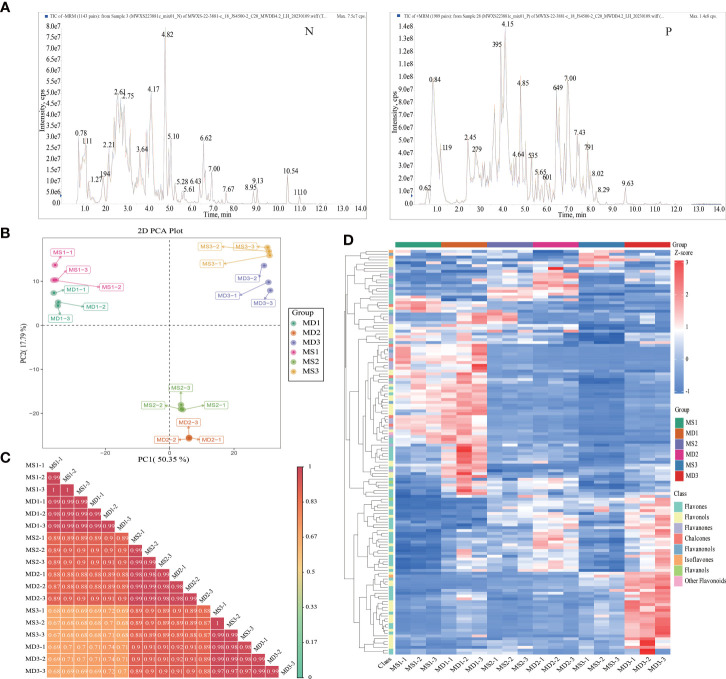
Profiling of flavonoid metabolites in MS and MD peels under magnesium stress. **(A)** Mixed sample mass spectrometry analysis total ions chromatogram. The horizontal axis represents the retention time of metabolite detection. The vertical axis represents the ion flow intensity of ion detection. N represents negative ion mode. P represents positive ion mode. **(B)** PCA plot of 18 samples based on flavonoid abundances. **(C)** Correlation analysis from 18 samples. **(D)** Heatmap of favonoids metabolites. High and low abundance are indicated by red and blue colors, respectively.

Based on an extensive target secondary metabolomics approach using UPLC-MS/MS analysis, significant differences were observed in flavonoid metabolites in SOPs at different developmental stages (**Figure 3A**). A total of 1,533 metabolites were detected from both MS and MD peel samples (**Supplementary Table 2**). All secondary metabolites were divided into nine classes, with 740 flavonoids being the most abundant (48.27%) (**Supplementary Figure 1**). The results strongly suggest that magnesium stress had a profound effect on the flavonoid metabolic pathway.

The first two principal components accounted for 50.35% (PC1) and 17.79% (PC2), respectively, and the 18 samples (including 3 replicates) were classified into 6 groups based on their developmental stage along PC1. The sample positions along PC2 were influenced by magnesium stress (**Figure 3B**). These findings suggest that the observed differences in flavonoid profiles were related to developmental stages and magnesium stress and were consistent with the trend in total flavonoid accumulation that peaked in MS2 or MD2 (**Figures 3B, C**). In addition, OPLS-DA analysis was utilized to evaluate the differences between MS and MD (Q2 = 0.99) (**Supplementary Figure 2**). The high Q2 value (>0.9) suggested that the OPLS-DA modules were stable and reliable and that the differences in flavonoid content could be further explored. Hierarchical clustering analysis (HCA) of the flavonoid metabolite accumulation patterns among different samples showed good repeatability within the sample group (**Figures 3D**). In the HCA, six clusters, corresponding to the successive stages of flavonoid metabolites in SOPs for the 18 samples, were significantly separated. The results of PCA, OPLS-DA, correlation analysis, and HCA reflected large differences between samples, high similarity among the three biological replicates, and high repeatability within samples.

### Differentially accumulated flavonoids metabolites in SOPs

**Figure 4:**
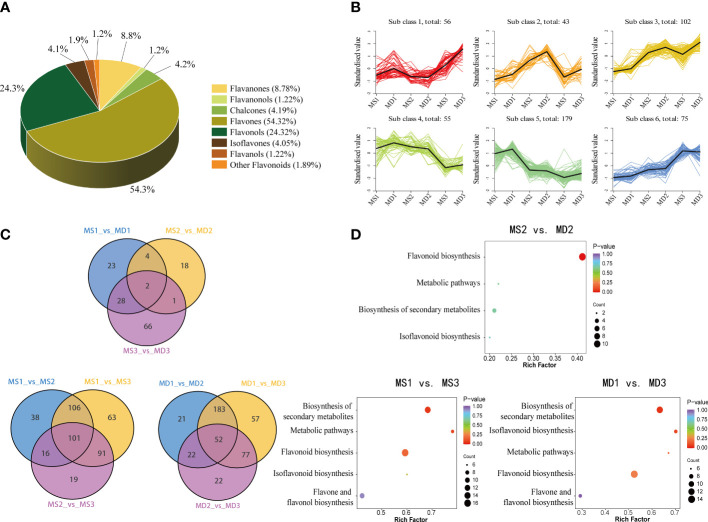
Analysis of differentially flavonoid metabolites in MS and MD peels under magnesium stress. **(A)** Classifications and proportions of 740 flavonoids detected in SOPs. **(B)** Trend analysis of differentially metabolics in SOPs. **(C)** Venn diagrams of DAFs. **(D)** KEGG enrichment analysis of DEGs in MS2 vs. MD2, MS1 vs. MS3, and MD1 vs. MD3.

According to modifications to the C3-C6-C3 structure, the 740 flavonoids were classified into eight classes: 402 flavones, 80 flavonols, 65 flavanones, 31 chalcones, 30 isoflavones, 9 flavanols, 9 flavanonols, and 14 other flavonoids (**Figure 4A**; **Supplementary Table 3**). Among these, limocitrin5-[6’’-(3-Hydroxy-3-Methylglutaryl) Glucoside] was the most prevalent flavonoid in SOPs, followed by limocitrin-3-O-(3-hydroxy-3-methylglutarate) glucoside, gardenin C malonyl glucoside, sinensetin, nobiletin, 5-Demethoxynobiletin, and 3’,4’,6,7,8-Pentamethoxyflavone (**Supplementary Figure 3**). Kmeans analysis revealed that all flavonoid compounds could be divided into six clusters (**Figure 4B**). Notably, the variation trend of two of the six clusters (class 2 and class 3) was consistent with the total flavonoid content. Moreover, there were significant differences in flavonoids compounds between MS and MD at different developmental stages, which might account for the disparity in total flavonoid content. Cluster 2 and cluster 3 had the highest proportion of flavones (98) (**Supplementary Figure 4**), suggesting that these clusters might play a critical role in flavonoid biosynthesis under magnesium stress in SOPs, with flavones significantly affected.

An investigation of differentially accumulated flavonoids (DAFs) was conducted in SOPs at different development stages. A total of 740 flavonoids were screened, and 142 DAFs were selected based on a fold change of |log2FC| ≥ 2 or |log2FC| ≤ 0.5 and a variable importance in projection (VIP ≥1) (**Supplementary Table 4**). Of these, 57 DAFs were identified in MS1 vs. MD1, followed by MS2 vs. MD2 (25) and MS3 vs. MD3 (97) (**Figure 4C**). The Venn Diagram results revealed two common and unique differential metabolites (Chrysoeriol-7-O-glucoside, Chrysoeriol-7-O-(6’’-feruloyl) glucoside) between MS and MD across all three periods. These flavonoids were flavones, and their change trend was consistent with total flavonoid content. To study the variation of these differential metabolites under magnesium stress, volcano diagrams were performed (**Supplementary Figure 5**). The results indicated that there were more up-regulated than down-regulated flavonoids in three stages between MS and MD. Specifically, 57 DAFs (53 upregulated and 4 downregulated) were identified during MS1 vs. MD1, 25 DAFs (6 upregulated and 19 downregulated) were identified during MS2 vs. MD2, and 97 DAFs (86 upregulated and 11 downregulated) were identified during MS3 vs. MD3. The majority of DAFs were observed during the development period. There were 278 DAFs (141 upregulated and 137 downregulated) and 261 DAFs (161 upregulated and 100 downregulated) selected from MD1 vs. MD2 and MS1 vs. MS2, respectively. The greater number of DAFs in MD than MS suggested that flavonoids may have been more susceptible to magnesium stress. The interaction of DAFs in SOPs resulted in the formation of different pathways, which were annotated and assigned to the KEGG pathways (**Figure 4D**). KEGG pathway enrichment analysis showed that flavonoid biosynthesis, phenylpropanoid biosynthesis, flavone and flavonol biosynthesis, secondary metabolites biosynthesis and metabolic pathways were the main enrichment pathways. Therefore, it could be postulated that the differentially accumulated metabolites (DAMs) in the pathways mentioned above may contribute to the variation in flavonoids of SOPs during the developmental process.

### Differentially expressed gene analysis

**Figure 5:**
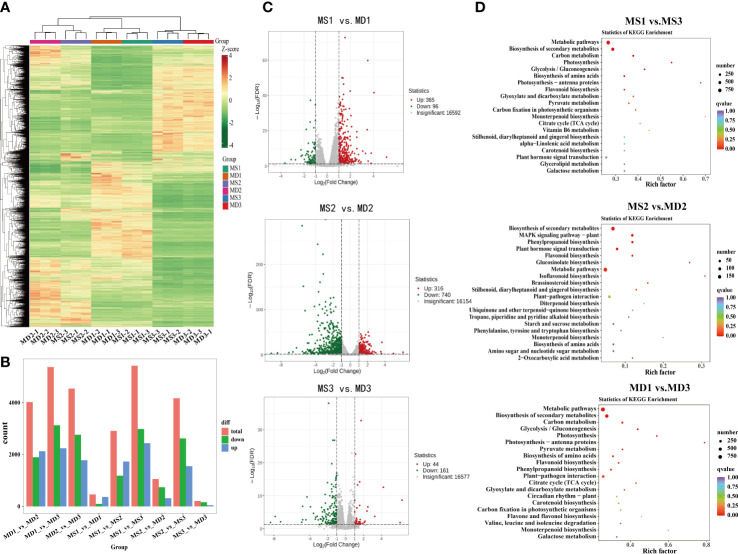
Analysis of differentially expressed genes (DEGs) and functional annotation in SOPs under magnesium stress in three stages. **(A)** Heat map cluster analysis of DEGs. **(B)** Number of DEGs in different groups. **(C)** Volcano plots for DEGs for MS1 vs. MD1, MS2 vs. MD2, MS3 vs. MD3, respectively. **(D)** KEGG enrichment analysis of DEGs in MS1 vs. MS3, MS2 vs. MD2, and MD1 vs. MD3. Dot size represents the number of distinct genes, and dot color reflects the q-value.

To further investigate the potential molecular mechanisms underlying flavonoid synthesis in the development of SOPs between MS and MD, 18 cDNA libraries were constructed for RNA-seq analysis. All transcriptional data were deposited in the NCBI Sequence Read Archive (BioProject: PRJNA934884). After filtering raw data, sequencing error rate, and GC content distribution inspection, the 18 libraries produced 140.33Gb of clean data, with Q30 percentages (percentage of sequences with sequencing error rates <0.03%) and GC percentages ranging from 92.29% to 93.22% and 43.15% to 43.65%, respectively (**Supplementary Table 5**). The 18 sequencing libraries were named as MS1-1, MS1-2, MS1-3, MS2-1, MS2-2, MS2-3, MS3-1, MS3-2, MS3-3, MD1-1,MD1-2,MD1-3, MD2-1,MD2-2,MD2-3, MD3-1,MD3-2, and MD3-3. In total, 15,730, 22,846, 29,125, 19,489, 27,941, 25,203, and 21,187 unigenes were annotated on KEGG, GO, NR, SwissProt, Trembl, KOG and Pfam databases. These results indicated that RNA-seq was of high quality and suitable for further analysis. A total of 17,897 differentially expressed genes (DEGs) were detected in three stages (**Supplementary Table 6**). Heat map cluster analysis showed significant differences in gene expression between different treatments (**Figure 5A**). In SOPs, 461 DEGs were identified in MS1 vs. MD1, followed by MS2 vs. MD2 (1056) and MS3 vs. MD3 (205) (**Figure 5B**). In addition, 861 and 715 DEGs were identified in three stages of MS and MD, respectively (**Supplementary Figure 6**). There were large numbers of DEGs between MD and MS at each stage, and more upregulated DEGs than downregulated DEGs were found in MS1 vs. MD1, but fewer upregulated DEGs were present in MS2 vs. MD2 and MS3 vs. MD3 (**Figure 5C**), indicating that genes might have different response patterns to magnesium stress at different developmental stages.

In GO annotation analysis, a total of 38,397 DEGs were annotated in three categories: biological process, molecular function, and cellular component categories (**Supplementary Table 7**). These DEGs were further divided into 47 categories based on gene function, with 722 genes related to biological processes such as signaling. TopGO analysis revealed that the most enriched molecular function terms were monooxygenase activity (GO0004497), oxidoreductase activity (GO0016705), heme binding (GO0020037), iron ion binding (GO0005506), and tetrapyrrole binding (GO0046906) (**Supplementary Figure 7**). The most enriched biological process terms were flavonoid metabolic process (GO0009812) and flavonoid biosynthetic process (GO0009813). The most enriched cellular component terms were chloroplast thylakoid (GO0009534) and plastid thylakoid (GO0031976). KEGG enrichment analysis identified the top 20 enriched metabolic pathways, including metabolic pathways (ko01100), biosynthesis of secondary metabolites (ko01110), MAPK signaling pathway-plant (ko04016), plant hormone signal transduction (ko04075), phenylpropanoid biosynthesis (ko00940), and flavonoid biosynthesis (ko00941) under magnesium stress (**Figure 5D**). These results were presented in a bubble diagram.

### Metabolic and gene co-expression networks in SOPs at different developmental stages

**Figure 6:**
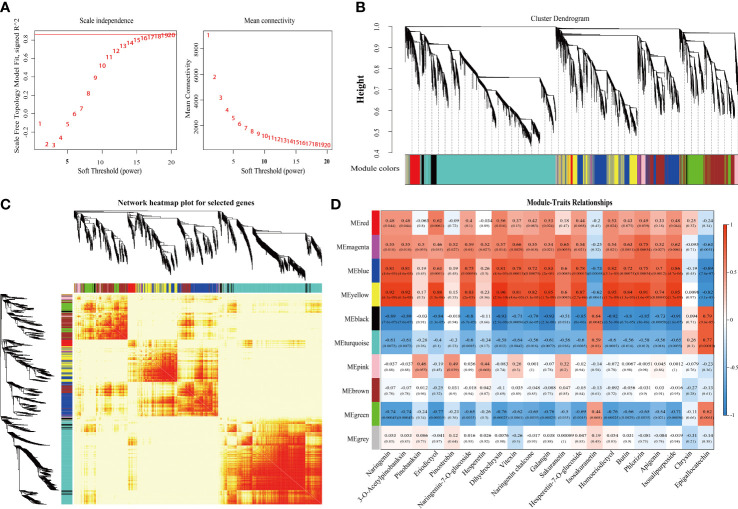
Integrative analysis of DEGs and DAMs under magnesium stress in MS and MD. **(A)** Determination of soft threshold. The abscissa represents the soft threshold (β). Ordinate corresponds to the index of scale free network model. The average link degree of each soft threshold. **(B)** Network heatmap plot of genes subjected to co-expression module calculation. **(C)** Module heatmap. **(D)** Module-trait relationship heat map. Red indicates high correlation, blue indicates low correlation.

To construct the co-expression network, low-expressed genes (FPKM value ≤ 0) were filtered and screened based on the expression matrix of unigenes from 18 samples, resulting in a total of 17,897 differentially expressed unigenes for WGCNA analysis (**Supplementary Table 6**). A soft threshold (power) of 20 was selected to construct the co-expression network (**Figure 6A**), and gene cluster trees were constructed to represent a cluster of genes whose expression levels were highly correlated. This approach resulted in the identification of nine modules labeled with different colors in MS and MD (**Figures 6B, C**), with each module containing DEGs with similar expression patterns. Genetic expression patterns varied between modules in MS and MD during three development stages. The heat map of the module-trait relationship was performed based on the DEGs combined with 21 DAFs in SOPs under magnesium stress (**Figure 6D**). The yellow module was found to be associated with 15 flavonoids and had the highest correlation with seven flavonoids (r > 0.9, p < 0.001). The bule module was positively correlated with the contents of naringenin (flavanones) and pinobanksin (flavanonols) (r = 0.81, p < 0.001), isosalipurposide (chalcones) (r = 0.86, p < 0.001), and naringenin chalcone (chalcones) (r = 0.83, p < 0.001). The gene expression pattern varied between the yellow and blue modules, which was visualized in a heatmap combined with a column chart (**Supplementary Figure 8**). Interestingly, the expression levels of most genes in the yellow module were increased under magnesium stress, and the expression levels of MD were higher than that of MS. This was consistent with the varied trend in total flavonoid content. GO analysis of the yellow module showed that its DEGs were significantly enriched in metabolic process and cellular process in the biological process category, and binding and catalytic activity in the molecular function category. KEGG enrichment analysis revealed that more than 40 DEGs were enriched in phenylpropanoid biosynthesis, isoflavonoid biosynthesis, flavonoid biosynthesis, and flavone and flavonol biosynthesis in yellow module genes (**Supplementary Figure 9**).

**Figure 7:**
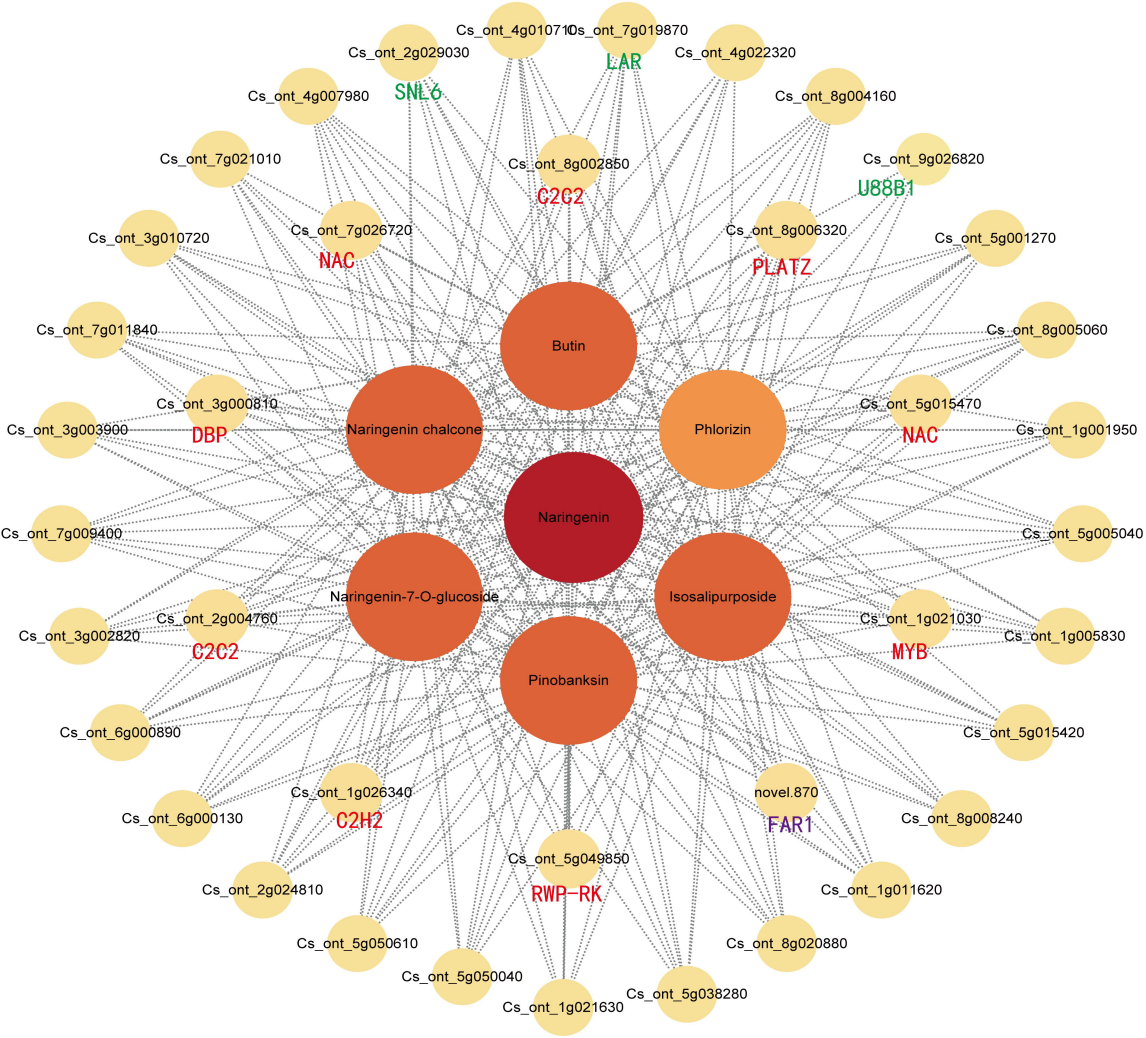
Weighted gene co-expression network analysis of DEGs related to flavonoid content under magnesium stress in the yellow modules. Green and purple represent structural genes and transcription factors that have been annotated and identified by KEGG, respectively. Red represents unidentified transcription factors with a correlation greater than 0.95.

The gene co-expression network was constructed based on the strong positive correlation with flavonoid content in the yellow module (**Figure 7**). The network diagram identified three structural genes [1 *CitLAR* (Cs_ont_7g019870), 1 *CitU88B1* (Cs_ont_9g026820), 1 *CitSNL6* (Cs_ont_2g029030)] involved in the flavonoid biosynthesis pathway. In the interaction network diagram, 10 transcription factor (TF) genes formed the outer layer consisted with strong correlations (above 0.95) with six flavonoids. Among these TF genes, there were two *C2C2-Dofs*, two *NACs*, one *PLATZ*, one *MYB*, one *C2H2*, one *FAR1*, one *RWP-RK*, and one *DBP*) (**Supplementary Table 8**). Similarly, the blue module contained three structural genes [2 *CitF3*’*H* (Cs_ont_2g033020, Cs_ont_9g024120), 1 *CitCHS* (Cs_ont_3g009610)] involved in flavonoid synthesis. Therefore, the network identified six structural genes and ten TF genes as key regulators of flavonoid accumulation in SOPs. These findings suggest that magnesium stress affected the transcription level of flavonoid biosynthesis genes, with significant differences between MD and MS. To validate the expression patterns of flavonoid-related genes, qRT-PCR was performed on six hub structural genes and ten hub TF genes from the WGCNA co-expression network (**Supplmentary Figure 10**). The results confirmed the accuracy and repeatability of the transcriptome data.

### Differentially expressed genes associated with flavonoids biosynthesis in SOPs

**Figure 8:**
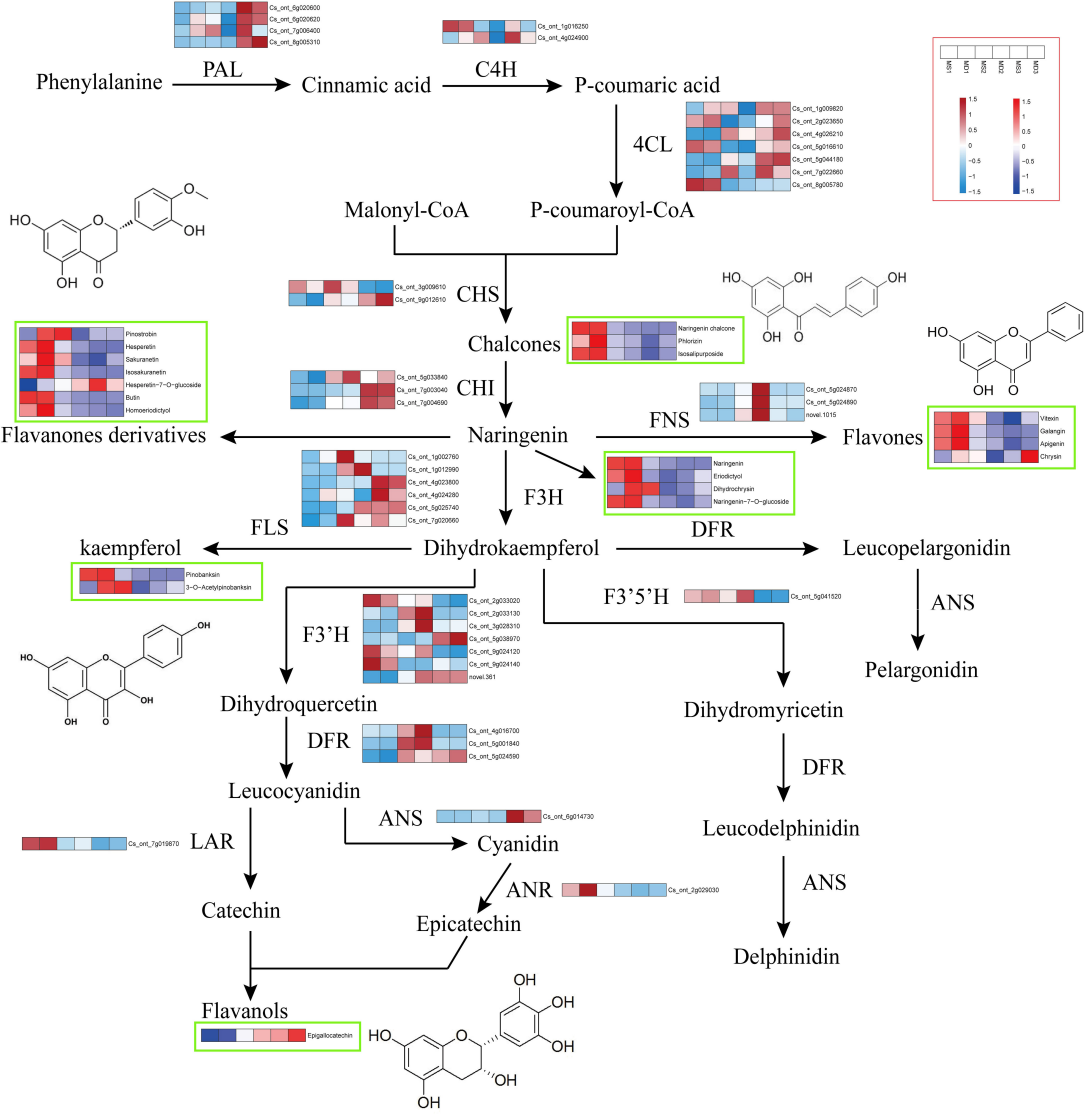
Flavonoid biosynthesis pathway in SOPs from MS and MD under magnesium stress. Gene expression is displayed in heatmaps based on mean FPKM. Azure indicates low expression, and pink indicates high expression. Flavonoid content is shown in heatmaps based on abundance in the metabolite profile. Flavonoids with high abundance are indicated in red, and those with low abundance are indicated in blue. PAL, phenylalanine ammonia lyase. C4H, cinnamate 4-hydroxylase. 4CL, 4-coumaroyl CoA ligase. CHS, chalcone synthase. CHI, chalcone isomerase. FNS, flavone synthase. F3H, flavanone 3 β-hydroxylase. DFR, dihydroflavonol-4-reducatse. F3’H, flavonoid 3’-hydroxylase. F3’5’H, flavonoid 3’,5’-hydroxylase. FLS, flavonol synthase. LAR, leucoanthocyanidin reductase. ANS, anthocyanidin synthase. ANR, anthocyanidin reductase.

In order to investigate the variations in flavonoid biosynthesis in SOPs at different developmental stages, an integrated analysis of transcriptome and metabolomics data was performed. Based on the flavonoid biosynthetic pathway reported in model plants, a pathway diagram indicating the expression of structural genes and flavonoids in SOPs was constructed (**Figure 8**). A total of 41 structural genes and 21 flavonoids were mapped to the flavonoid biosynthetic pathway. 13 structural genes (4 CitPALs, 2 CitC4Hs, and 7 Cit4CLs) participated in the phenylpropanoid pathway, and 28 structural genes (2 CitCHSs, 3 CitCHI*s*, 3 CitFNSs, 6 CitFLSs, 3 CitDFRs, 1 CitANS, 1 CitLAR, 1 CitANR, 7 CitF3’Hs, and 1 Cit F3’5’H) participated in the flavonoid pathway. The expression levels of one Cit4CL (Cs_ont_2g023650) and one CitCHS (Cs_ont_9g012610) were found to be consistent with Phlorizin (Chalcones) and Isosalipurposide (Chalcones) in MS vs. MD, respectively. Flavanones, which were the core metabolites of the flavonoid pathway, can be converted into flavones by FNS and dihydrokaempferol (dihydroflavonol) by F3H. The expression levels of one CitPAL (Cs_ont_6g020620) and one Cit4CL (Cs_ont_1g009820) were consistent with the content of Pinostrobin (flavanones derivatives) in MS vs. MD. However, the content of flavones did not fully match *CitFNS* expression levels. The expression level of one CitFNS (Cs_ont_5g024890) was found to be consistent with the trend in total flavonoid content. The differences in flavonols and flavanols content in MS and MD were consistent with the expression levels of the corresponding enzyme genes. The expression levels of one CitFLS (Cs_ont_1g002760), one CitF3’H (Cs_ont_5g038970), and one CitLAR (Cs_ont_7g019870) were consistent with 3-O-Acetylpinobanksin and Epigallocatechin in MS vs. MD.

### Correlation analysis and canonical correlation analysis between flavonoids content and expression of synthesis-related genes

**Figure 9:**
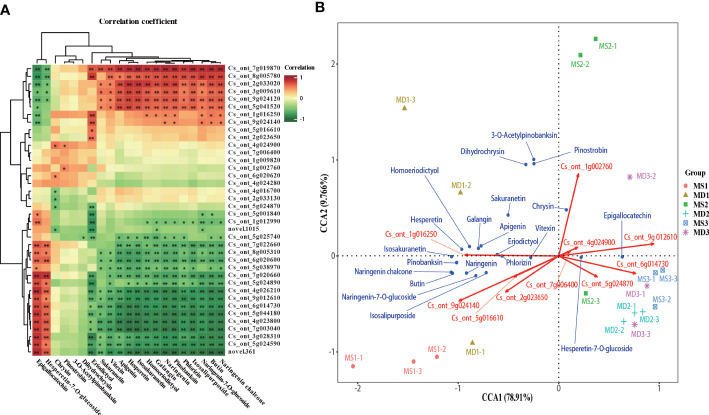
Screening of the main contributing genes for flavonoid biosynthesis in SOPs. **(A)** Intergroup correlation analysis of 21 flavonoids and 41 flavonoid synthesis–related genes. **(B)** Canonical correlation analysis (CCA) of 21 flavonoids and related synthetic genes between MS and MD in SOPs. *p < 0.05 and **p < 0.01.

Correlation analysis between the expression levels of 41 genes and the content of 21 flavonoids showed that Epigallocatechin and Hesperetin-7-O-glucoside significantly positively correlated with *CitCHI* (Cs_ont_7g003040) and one *CitCHS* (Cs_ont_9g012610), and 15 flavonoids significantly positively correlated with one *CitCHS* (Cs_ont_3g009610) (**Figure 9A**). To comprehensively analyze the variables, canonical correlation analysis (CCA) was used to analyze the relationship between flavonoids and synthetic pathway genes and screen the key genes for flavonoid biosynthesis in SOPs (**Figure 9B**). The dimensionality reduction of 41 genes in the flavonoid synthesis pathway was performed using CCA. The results showed that 10 genes played a significant role in the flavonoid biosynthesis of SOPs, including *Cit*PAL, *Cit*C4H, *Cit*4CL, *Cit*FNS, *Cit*FLS, *Cit*F3’H, and *Cit*ANS. The CCA results showed that the overall interpretation rate of the independent variable matrix (genes) to the dependent variable matrix (flavonoids) was 88.676% (CCA1 + CCA2), indicating that the analysis results were highly reliable. These results showed a correlation between the 10 major genes involved in flavonoid biosynthesis and the 21 flavonoids. Genes were divided into two groups according to their spatial distribution. The first group included *Cit*PAL (Cs_ont_7g006400), *Cit*4CL (Cs_ont_2g023650, Cs_ont_5g016610), *Cit*FNS (Cs_ont_5g024870), *Cit*ANS (Cs_ont_6g014730), and *Cit*F3’H (Cs_ont_9g024140), and the second group included *Cit*C4H (Cs_ont_4g024900, Cs_ont_1g016250), *Cit*CHS (Cs_ont_9g012610), and *Cit*FLS (Cs_ont_1g002760) (**Figure 9B**). A significant positive correlation was observed between genes within a group, while a negative correlation was found between genes in different groups. In MD1, the expression levels of *Cit*C4H (Cs_ont_1g016250) were significantly correlated with the contents of 3-O-Acetylpinobanksin, Pinostrobin, Sakuranetin, Homoeriodictyol, Hesperetin, Apigenin, Galangin, and Dihydrochrysin. In MS1, the expression levels of *Cit*4CL (Cs_ont_2g023650, Cs_ont_5g016610) and *Cit*F3’H (Cs_ont_9g024140) were significantly associated with the contents of pinobanksin, naringenin chalcone, naringenin, butin, naringenin-7-O-glucoside, and isosalipurposide). In MD2, MD3 and MS3, CitFNS (Cs_ont_5g024870) and CitANS (Cs_ont_6g014730) expression levels were significantly correlated with the contents of hesperetin-7-O-glucoside and epigallocatechin. In MS2, the expression levels of *Cit*FLS (Cs_ont_1g002760), *Cit*C4H (Cs_ont_4g024900) and *Cit*CHS (Cs_ont_9g012610) were significantly correlated with the contents of chrysin. The results indicated that magnesium stress had a significant influence on flavonoids at different stages of fruit growth and development.

## Discussion

Citrus peel is known for its high nutritional and medicinal value, attributed to its richness in flavonoids, which act as reducing agents in the antioxidant system of plants under abiotic stress (Ahmed et al., 2017; Nair et al., 2018; Shen et al., 2022). Flavonoids are the most abundant secondary metabolites in citrus fruits, and their accumulation is generally influenced by stress, such as drought, light, UV and iron stress (Heijde and Ulm, 2012; Page et al., 2012). Magnesium stress is a common environmental challenge for fruits, affecting their quality and yield, especially in areas with excessive use of chemical fertilizers and inadequate use of organic fertilizers and medium and trace element fertilizers (Liu et al., 2022). According to leaf nutrient classification standards, a Mg content ranging from 200 mg/kg to 290 mg/kg may represent Mg deficiency, and more than 300 mg/kg may represent Mg sufficiency in leaf (Collins et al., 2020). The magnesium content in MD leaves was less than 300mg/kg, indicating a magnesium deficiency, while the magnesium content in MS leaves was higher than 300mg/kg, indicating an appropriate magnesium level (**Figure 2C**). This was consistent with our expected judgment. Magnesium had high fluidity in older leaves and was transferred from leaf to fruit during citrus fruit development (Hauer-Jakli and Trankner, 2019). Therefore, magnesium deficiency was first manifested in the leaves. In this research, flavonoid content was found to be higher in MD under magnesium stress during three periods (**Figure 2D**), indicating that magnesium stress induced the accumulation of ROS and subsequently promoted the accumulation of flavonoids to protect plant cells from oxidative damage. In contrast, the total flavonoid content decreased in MS. Recent studies have found that a magnesium deficiency in leaves may affect fruit growth and development, accumulation of dry matter, and nutrient absorption (Kwon et al., 2019; Liu et al., 2022). This explained the significant difference in the content of flavonoids between MD and MS. Malondialdehyde (MDA) is an indicator of lipid peroxidation of plant cell membrane, indicating the strength of response to stress conditions, while superoxide dismutase (SOD) is an important reactive oxygen defense enzyme *in vivo*, which can reduce lipid peroxidation and membrane damage, and plays a protective role in cells (Siddique et al., 2012). The contents of MDA and SOD were found to be higher when citrus fruits were under magnesium stress (**Figures 2E, F**), suggesting that the ROS-scavenging system was activated, including oxidative enzymes such as SOD, as well as membrane lipid peroxidation substances such as MDA. MDA content and SOD activity trends were consistent with total flavonoid content. In the MS environment, SOPs might have reduced the accumulation of ROS by strengthening the antioxidant system, leading to lower ROS levels, obviating the need to activate flavonoid biosynthesis. Moreover, the precursors of flavonoid biosynthesis might also participate in other pathways, leading to reduced efficiency of flavonoid accumulation and lower flavonoid levels in MS. These findings highlight the importance of magnesium fertilization in citrus production.

Mass spectrometry-oriented metabolomics has emerged as a powerful tool for biological research, enabling the systematic identification and quantification of metabolites (Alseekh et al., 2021). In this study, a widely targeted metabolomic approach combined with transcriptome data was used to explore flavonoid accumulation and its underlying molecular regulation in SOPs under magnesium stress. Although previous studies had explored the flavonoid pathway in citrus, the number of flavonoid components identified remained limited (Yu et al., 2015; Wang et al., 2019; Yu et al., 2022). In our study, we identified 740 flavonoid components in SOPs, representing a significant supplement to the previous work. These flavonoids were classified into eight categories, with flavones (54.32%) being the most abundant class, followed by flavonols and flavanones (**Figure 4A**). These results indicated that they are the main flavonoid classes in SOPs and this was consistent with previous research (Wang et al., 2019). Flavonoid carbonosides, or flavonoid glycosides, which are stable forms of flavonoids with sugar groups bound to an aglycone carbon, were also identified in high numbers (277) in SOPs. These compounds are important phytochemicals in the human diet and have been reported as active components of traditional Chinese medicines with various medicinal properties (Shen et al., 2022), including anti-inflammatory and antioxidant activities (Matsia et al., 2022; Wang et al., 2022). The content of Chrysoeriol-7-O-glucoside and Chrysoeriol-7-O-(6’’-feruloyl) glucoside were found to be higher in MD compared to MS during all three stages. In addition, the combined content of all flavonoid carbonosides in MD was also greater than that of MS, which correlated with the overall trend in total flavonoid content (**Figure 2C**). SOPs under magnesium stress showed higher contents of flavonoid carbonosides, suggesting their potential for functional food production and as a source of bioactive compounds for medication. Furthermore, SOPs also showed higher levels of flavanols, with (-)-Epicatechin-(4β->8)-(-)-epigallocatechin as the predominant component. This suggests that mandarin orange peels under magnesium stress may be a potential source for natural functional beverages and oral liquids.

Under environmental stress, plant cells activate gene expression programs at the transcriptional level, regulating metabolite accumulation to adapt to the new conditions (Howarth and Ougham, 1993). Transcriptome data can provide important insights into the key genes involved in targeted pathways. In this study, transcriptome analysis was used to uncover the molecular mechanisms governing flavonoid content in SOPs under magnesium stress. GO annotation and KEGG enrichment analyses highlighted potential biological processes and pathways that played a role in the accumulation and transport of primary and secondary metabolites, including flavonoids. WGCNA was used to identify genes with similar expression patterns, resulting in the identification of a yellow module that showed a significant correlation with seven flavonoid contents. Six structural genes and ten TFs were identified as being highly correlated with flavonoid biosynthesis. Together, these 16 hub genes were considered to be major regulators of flavonoid biosynthesis in SOPs under magnesium stress. The flavonoid biosynthesis pathway has been elucidated in some model plants, where chalcone is synthesized from phenylalanine through the phenylpropanoid pathway, with PAL, C4H, 4CL, and CHS serving as key rate-limiting enzymes. As previously reported, overexpression of *CitCHI*L1 significantly increased the content of total flavanones and flavones, while virus-induced gene silencing (VIGS) of CitCHIL1 led to a decrease in the total flavonoid content in Ougan (Citrus sinensis (L.) Osbeck) (Zhao et al., 2021). Chalcone synthase gene (*CHS*) and Chalcone isomerase gene (*CHI*) are the backbone genes in the flavonoid biosynthesis pathway, and their expression levels directly affect the synthesis of different flavonoids. For example, Hesperetin-7-O-glucoside had a significant negative correlation with *CHS* but played a positive role in synthesizing galangin. Meanwhile, CCA has been shown to have a wide range of applications in solving practical problems and has demonstrated a correlation between the two groups of indicators as a whole (Garcia et al., 2005). The results of this study showed that the expression level of one Cit*CH*S (Cs_ont_9g012610) was consistent with the content of one flavone (chrysin). Flavones were the main flavonoid compounds in citrus, and their synthetic structural genes and transcription factors have become the focus of attention. Therefore, *CitCHS* (Cs_ont_9g012610) was hypothesized to be a key gene for flavone and flavonoid synthesis in SOPs under magnesium stress.

Environmental factors can regulate gene expression by influencing TFs, which bind specifically to the promoters of their target genes. Among TF families, the *MYB* family has been shown to play a critical role in regulating gene expression in the flavonoid pathway (Espley et al., 2007; Zhou et al., 2015; Zhai et al., 2016; Li et al., 2020a). For instance, the MdBBX22–miR858–MdMYB9/11/12 was found to activate the promoters of MdANR and MdLAR in apple, thereby promoting the biosynthesis of proanthocyanidin (Zhang et al., 2022). In this study, one *CitMYB* (Cs_ont_1g021030) was identified as highly related to structural genes and seven flavonoids based on WGCNA. Therefore, these ten TF genes were considered important in regulating the flavonoid content of SOPs. Although the results of qRT-PCR showed good consistency with the transcriptome data (**Supplementary Figure 10**), future studies are needed to elucidate the function of these genes in flavonoid biosynthesis.

## Conclusion

**Figure 10:**
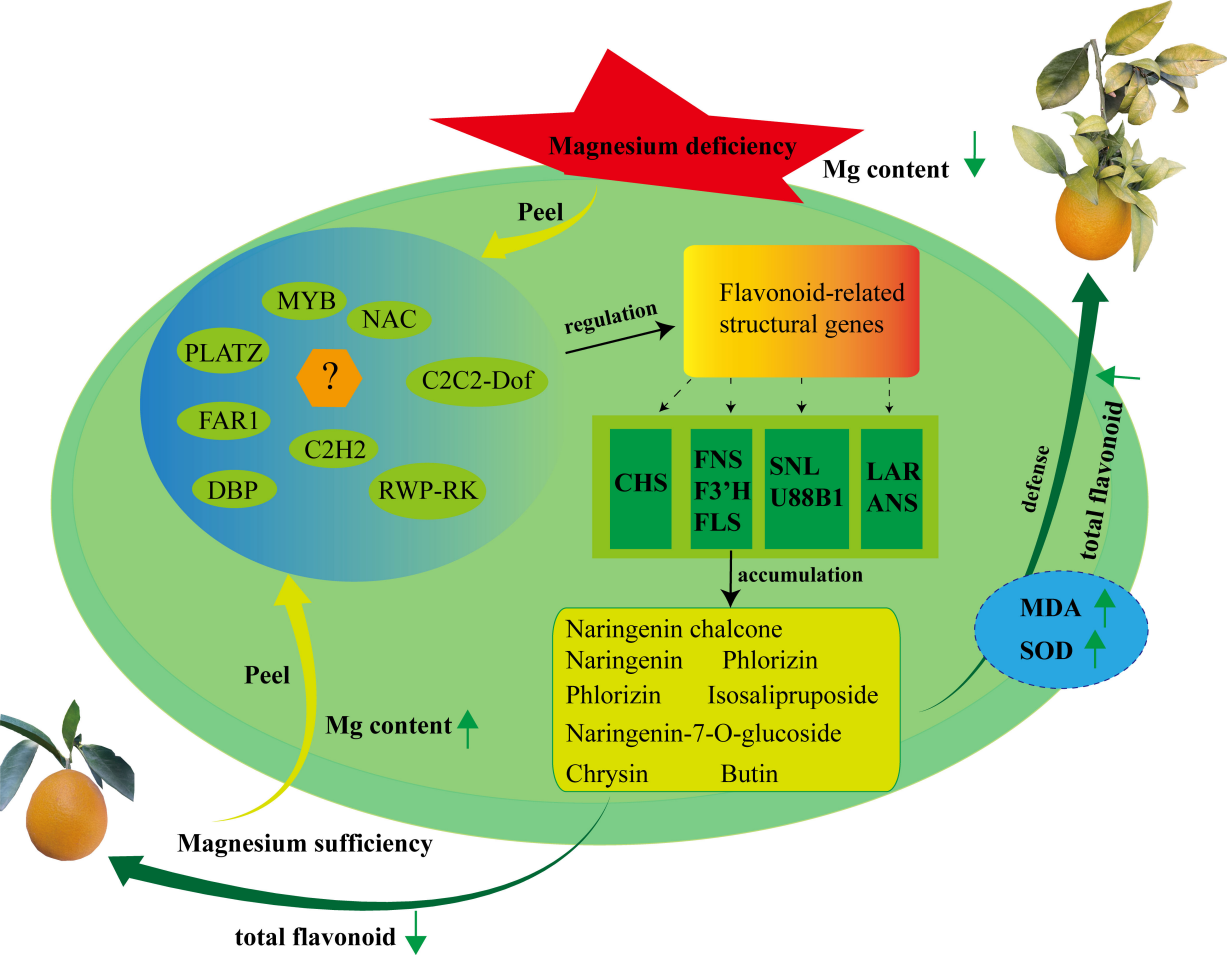
Proposed model for flavonoid biosynthesis in SOPs under magnesium stress. Hub genes and metabolites were identified in the trend analyses, WGCNA, heatmap and CCA. Widely targeted metabolomic profiling combined with transcriptome analysis revealed that flavonoid compounds synthesis was regulated by transcription factors and structural genes during fruit developmental period. Up arrows represent raising, and down arrows represent lowering.

In this study, 1,533 metabolites were identified in SOPs using UPLC-MS/MS. Among them were 740 flavonoids and their derivatives covering different groups such as 432 flavoens, 180 flavonols, 65 flavanones, 31 chalcones, 30 isoflavones, 9 flavanonols, 9 isoflavones, and 14 flavonoids carbonosides. Flavones were found to be the most abundant type of flavonoid present. The results showed that magnesium stress increased the total flavonoid and MDA content as well as SOD activity, indicating that magnesium-deficiency may enhance the nutritional value of flavonoids in SOPs. Using gene expression analysis, six flavonoid synthesis-related genes (*CitLAR*, *CitU88B1*, *CitSNL*, *CitCHS*, and 2 *CitF3’Hs*) and ten TFs genes were identified by WGCNA. Ten additional flavonoid synthesis-related genes were also identified by CCA and relation heatmap. *CitCHS* was found to play a key role in flavonoid synthesis under magnesium stress in SOPs (**Figure 10**). qRT-PCR results were consistent with the transcriptome data, validating the accuracy of both the transcriptome sequencing and the candidate genes. This study provides crucial information regarding the flavonoid composition of SOPs and sheds light on the molecular regulation of flavonoid accumulation under magnesium stress. The results could be useful for the development of food nutrition and provide important clues for the cultivation of high-flavonoid SOPs.

## Data availability statement

The original contributions presented in the study are publicly available. This data can be found here: NCBI Sequence Read Archive (BioProject: PRJNA934884).

## Author contributions

Project and experiment design, ZW and BX. Experiment execution, QL, JY, ZL, XXY, and XYY. Data analysis, QL, YL, LL, XW, HD, and MZ. Writing, BX and QL. Review, BX and ZW. Project management, BX, GS, and ZW. All authors contributed to the article and approved the submitted version.
