# Supplementary material for: Widely targeted metabolomic profiling combined with transcriptome analysis sheds light on flavonoid biosynthesis in sweet orange 'Newhall' (C. sinensis) under magnesium stress
Source: Front Plant Sci. 2023 May 12;14:1182284. doi: 10.3389/fpls.2023.1182284 (PMC10216496; doi:10.3389/fpls.2023.1182284)
Supplement: Supplementary file 1 [file DataSheet_1.pdf]

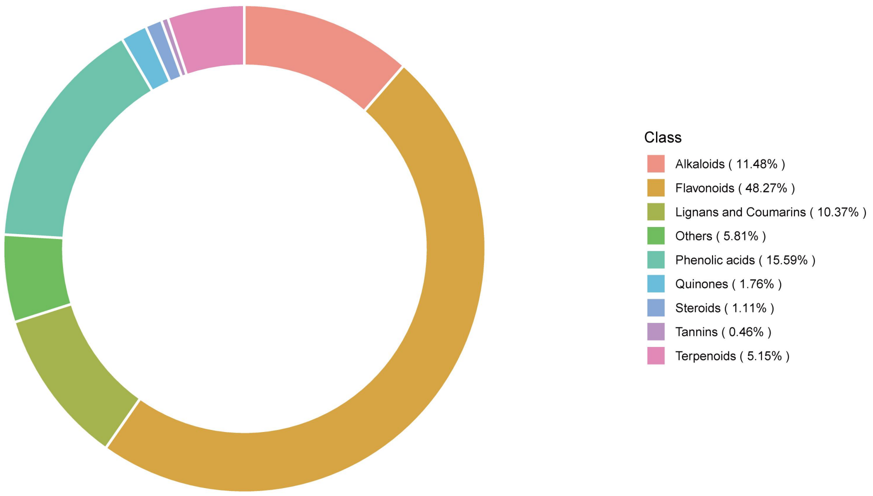

FIGURE S1: Classifications and proportions of 1533 metabolites detected in SOPs. The mean values and SDs were calculated using one-way ANOVA followed by Duncan's multiple range test.

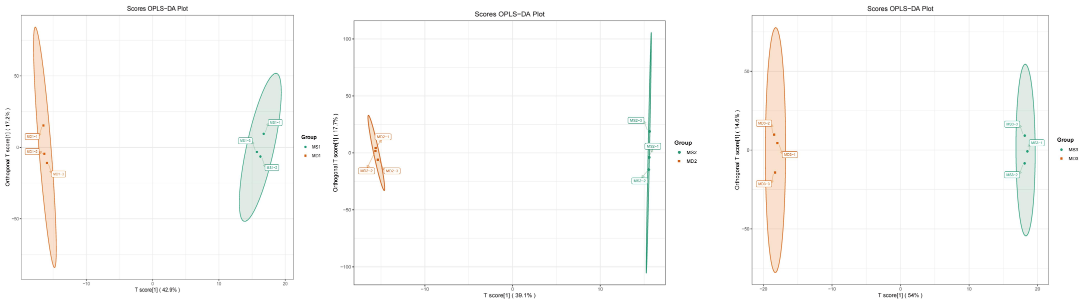

FIGURE S2: OPLS-DA plots of 18 samples in SOPs of MS1 vs. MD1, MS2 vs. MD2, and MS3 vs. MD3.

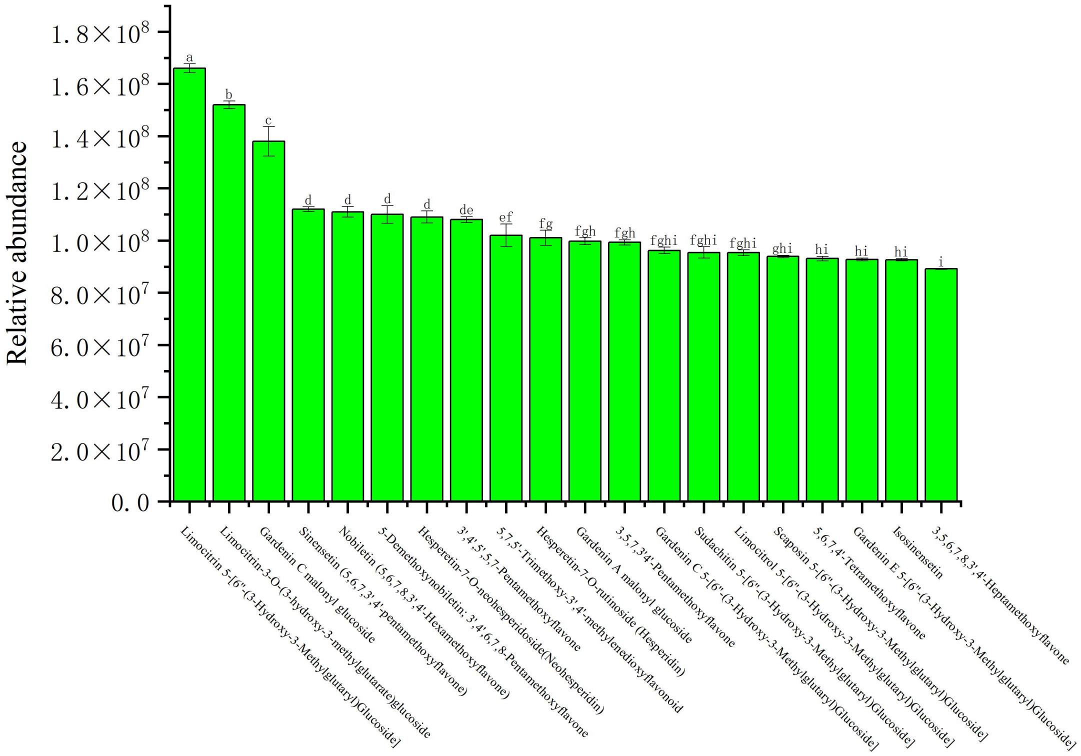

FIGURE S3: The top 20 most abundant flavonoid compounds in SOPs.

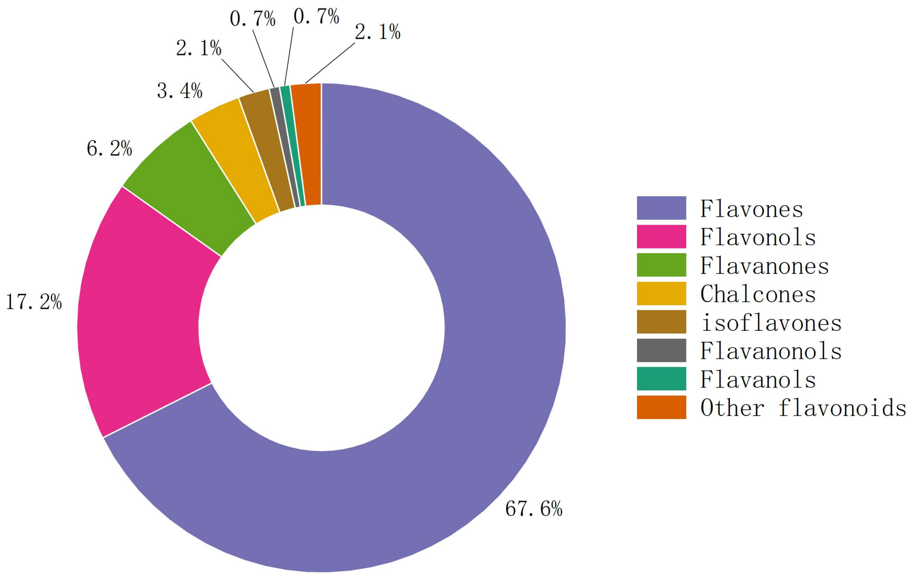

FIGURE S4: The proportion of flavonoids in clusters 2 and 3 in Kmeans.

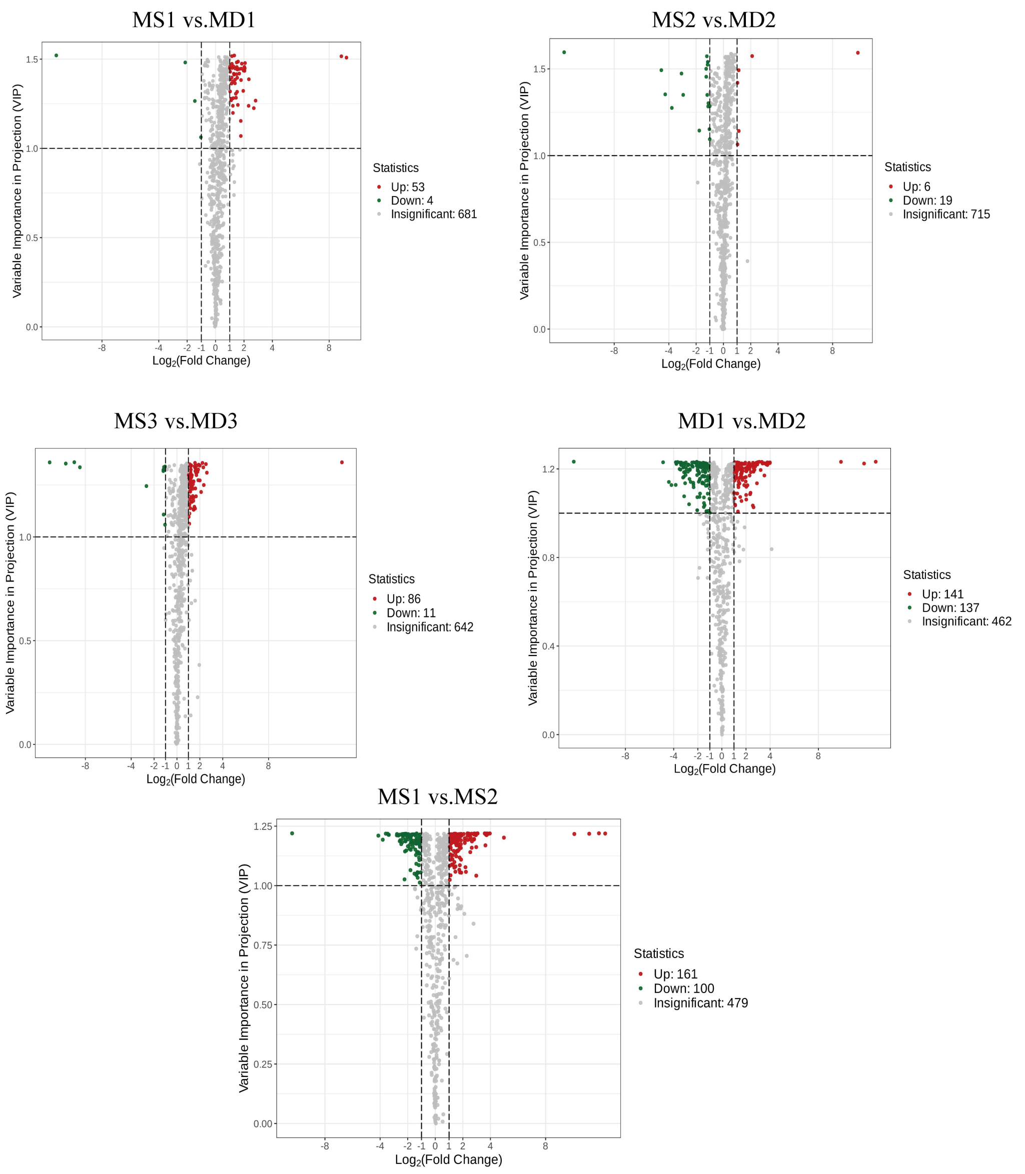

FIGURE S5: Volcano plots for DAFs for MS1 vs. MD1, MS2 vs. MD2, MS3 vs. MD3, MD1 vs. MD2, and MS1 vs. MS2 ,respectively.

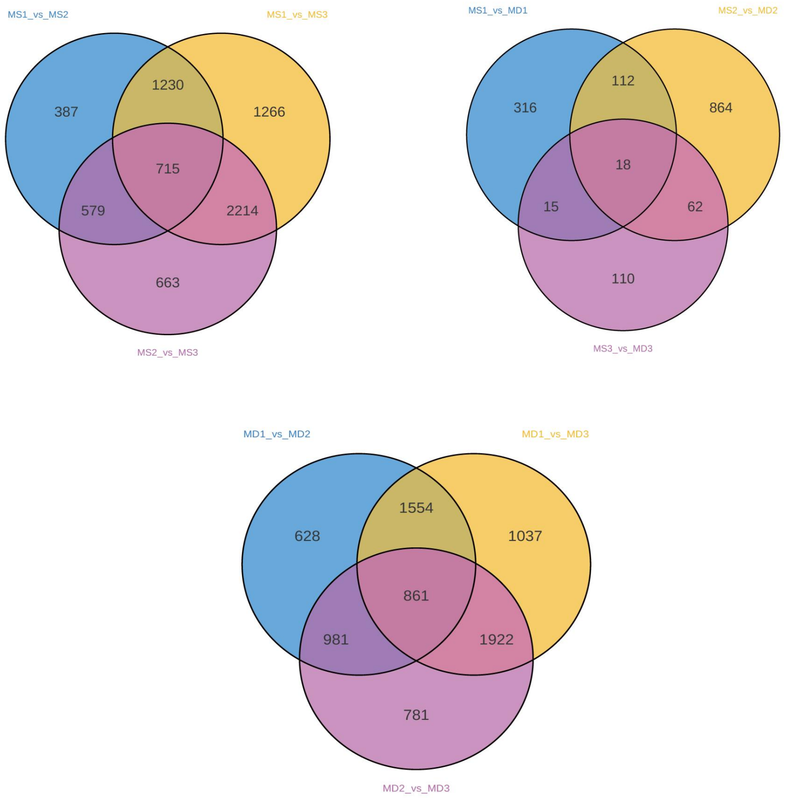

FIGURE S6: Venn diagrams of DEGs in MD, MS and MS vs. MD in SOPs during three stages.

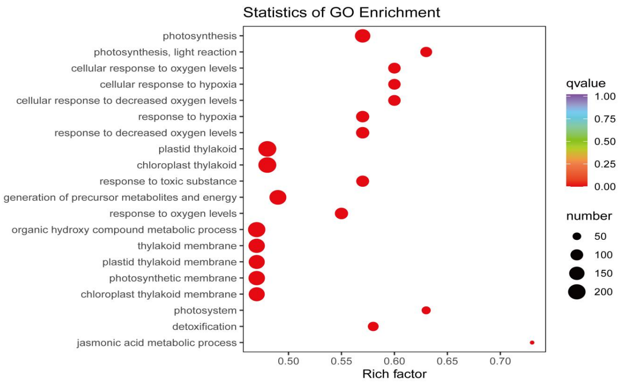

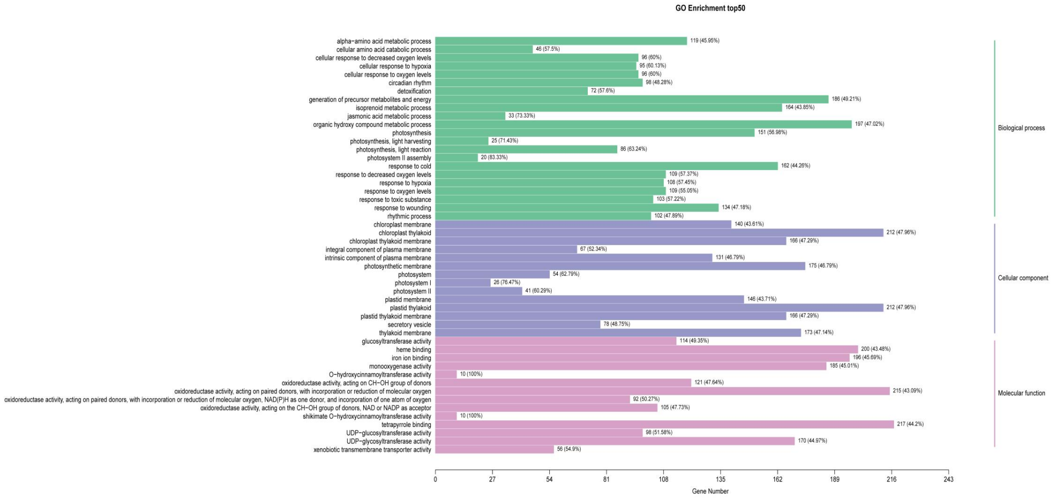

FIGURE S7: GO enrichment top 50 and GO classification.

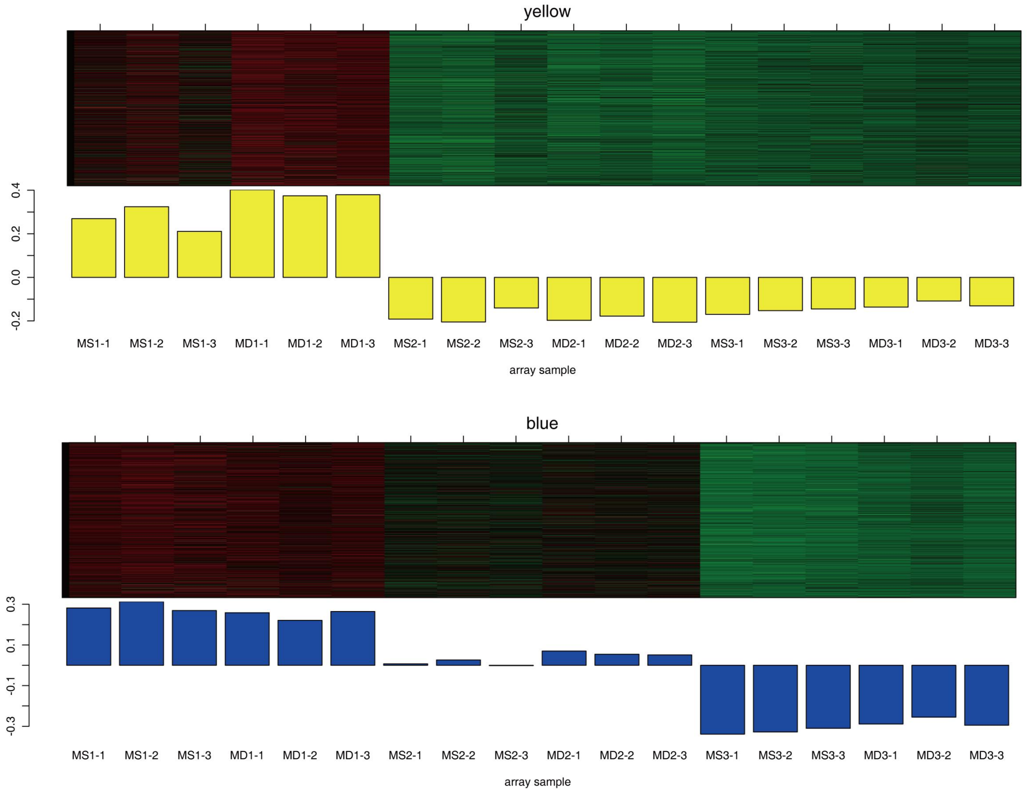

FIGURE S8: Eigengene expression profile for yellow and blue modules in different samples. The heat map above shows the expression profiles of all co-expressed genes. The bar graph below shows the common expression patterns of co-expressed genes.

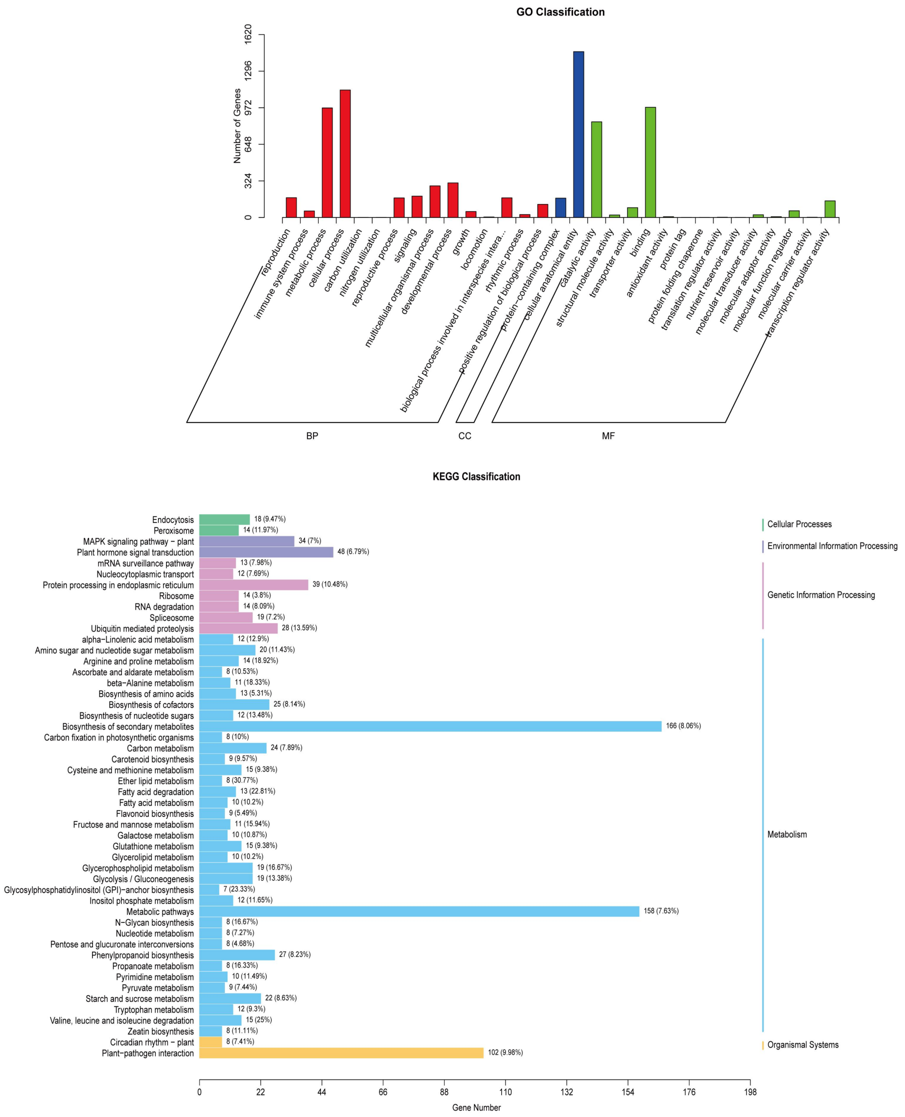

FIGURE S9: GO and KEGG in yellow module.

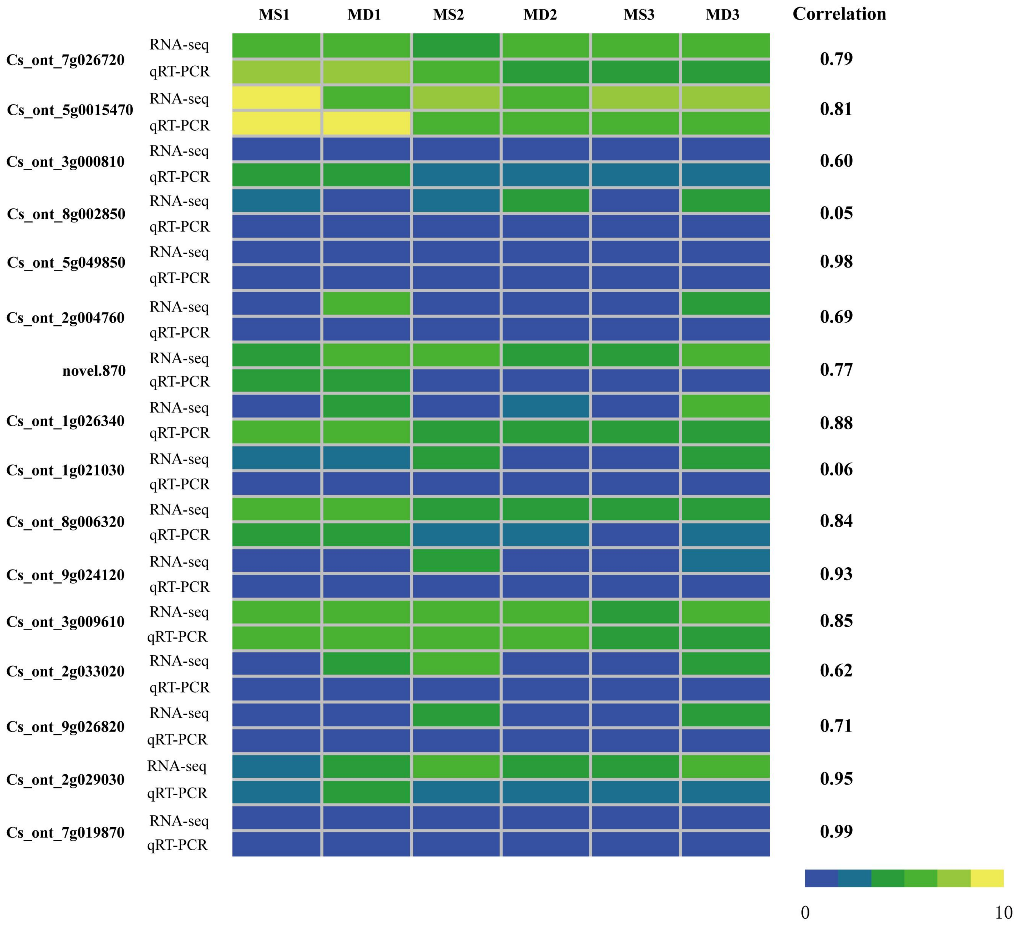

FIGURE S10: qRT-PCR analysis of hub genes in the co-expression network yellow and blue module.
